# Evaluating evidence regarding the efficacy of time-restricted eating for weight loss

**DOI:** 10.3389/fnut.2024.1428998

**Published:** 2024-06-27

**Authors:** David E. Most

**Affiliations:** ^1^School of Education, Colorado State University, Fort Collins, CO, United States; ^2^Department of Biostatistics and Informatics, Colorado School of Public Health, Fort Collins, CO, United States

**Keywords:** time-restricted eating, weight loss, scientific inference and reasoning, statistical inference abuse, meta-analytic thinking

## Introduction

The goal of this commentary is to reassess research findings shared by Liu et al. in The New England Journal of Medicine regarding the impact of time-restricted eating (1). Liu et al. reported on the results of a trial in which 139 patients with obesity were randomly assigned to either time-restricted eating with daily calorie restriction or only daily calorie restriction. These two groups of patients were followed up for 12 months, with a focus on the difference between the two groups in change from baseline on body weight. Secondary outcomes of interest included waist circumference, body mass index (BMI), body fat, and metabolic risk factors. In the Conclusion section of the Abstract, Liu et al. wrote that “time-restricted eating was not more beneficial.” In the Discussion section of their manuscript, Liu et al. wrote that “the 8-hour time-restricted eating regimen did not produce greater weight loss” and that “the two dieting regimens in our trial had similar efficacy in reducing the levels of body fat.” In the last sentence of their manuscript, they wrote that “we found that the two weight-loss regimens that we evaluated had similar success.” The key concern discussed here is that, for the outcomes of interest, the evidence seems inconsistent with such conclusions.

## Evidence and meaning-making

What does the evidence seem to indicate? For all outcomes of interest, point and interval estimates of mean change are clearly presented for both groups and the difference between groups. The mean weight loss for the time-restricted eating group was 8.0 kg, while it was 6.3 kg in the group without time restrictions. One way to interpret this difference is to note that the mean weight loss in the time-restricted eating group was 27% higher than that in the group without time restrictions. The mean reduction in body fat mass for the time-restricted eating group was 5.9 kg, compared to 4.5 kg in the group without time restrictions. In other words, the mean reduction in body fat mass was 31% higher in the time-restricted eating group compared to the group without time restrictions. Regarding waist circumference and BMI, the mean reduction for both outcomes was 26% higher in the time-restricted eating group compared to the group without time restrictions. Similarly, for metabolic risk factors, compared to the group without time restrictions, the time-restricted eating group had a mean reduction in triglycerides that was 30% higher, in glucose levels that were 17% higher, and in the Homeostatic Model Assessment of Insulin Resistance (HOMA-IR) index that was 100% higher. In short, for all these outcomes of interest, the time-restricted eating regimen *was* more beneficial.

What accounts for the inconsistency between the evidence and the meaning-making? The reason for the inconsistency is an error in interpretation that is quite common. The error arises when one makes a clinical interpretation of evidence-based primarily or exclusively on a binary statistical declaration. A statistical claim of “no significant difference” leads to a scientific conclusion of “no difference between groups.” The authors' meaning-making of their results is based on such statistical declarations rather than on clinical judgments regarding the magnitude of the absolute or relative differences between the groups. In short, “we should never conclude there is ‘no difference' or ‘no association' just because a *p*-value is larger than a threshold such as 0.05 or, equivalently, because a confidence interval includes zero” (2).

What about uncertainty in the estimates of the between-group differences? The inclusion of 95% confidence intervals (Cis) for the key quantities of interest in both the prose and the tables in the manuscript helps to provide a sense of the magnitude of uncertainty in estimates of group differences. However, Liu et al. do not utilize the continuous nature of the CIs when interpreting their findings. Rather, because the CIs include zero, they declare, in a binary way, that there was no significant difference between the groups. For example, for the key outcome of weight loss, the 95% CI for the between-group difference in weight change is (−4.0, 0.4). A consideration of this CI suggests that the plausible true values for between-group differences in weight loss range from approximately 0 to 4 kg. Embracing uncertainty means that, while it is plausible that time-restricted eating is not more beneficial, the range of plausible true differences between groups that are compatible with the data includes a variety of values, many of which would be considered clinically significant. The CIs for between-group differences in change in waist circumference, BMI, body fat, and metabolic risk factors warrant a similar interpretation. Recognizing the meaning of this uncertainty aligns with the perspective that the evidence from this trial is not consistent with a clinical conclusion that time-restricted eating was not beneficial.

## Discussion

The problem discussed here is a single example of the century-old problem of confusing statistical inference with scientific inference (3). The consequence of this sort of error is that meaning-making is compromised, and, unfortunately, such interpretational errors are common in a variety of disciplines (2). The interpretation that the two diet regimens in the trial resulted in similar success for patients with obesity depends on mistakenly conflating the notion of a decision regarding (statistical) significance with a clinical evaluation of the nature of a difference. Rather than focusing on a binary declaration regarding whether the true efficacy of time-restricted eating could be zero, a better way to make meaning of these data would be to evaluate the magnitudes of differences between the two groups on the key outcomes in the trial. This sort of evaluation would be driven by clinical expertise while simultaneously embracing statistical and scientific uncertainty. Knowledge generation and associated clinical recommendations depend on scientific summaries that are faithful to the evidence.

## Author contributions

DM: Writing – original draft, Writing – review & editing.
